# Generation of an inducible RPE-specific *Cre* transgenic-mouse line

**DOI:** 10.1371/journal.pone.0207222

**Published:** 2018-11-15

**Authors:** Sandra Schneider, Nathan Hotaling, Maria Campos, Sarita Rani Patnaik, Kapil Bharti, Helen Louise May-Simera

**Affiliations:** 1 Institute of Molecular Physiology, Johannes-Gutenberg University, Mainz, Germany; 2 National Eye Institute, NIH, Bethesda, MD, United States of America; University of Florida, UNITED STATES

## Abstract

The retinal pigment epithelium (RPE) is an epithelial monolayer in the back of the vertebrate eye. RPE dysfunction is associated with retinal degeneration and blindness. In order to fully understand how dysregulation affects visual function, RPE-specific gene knockouts are indispensable. Since the currently available RPE-specific Cre recombinases show lack of specificity or poor recombination, we sought to generate an alternative. We generated a tamoxifen-inducible RPE-specific *Cre* transgenic mouse line under transcriptional control of an RPE-specific *Tyrosinase* enhancer. We characterized the Cre-mediated recombinant expression by crossing our *RPE-Tyrosinase-CreEr*^*T2*^ mouse line with the *tdTomato* reporter line, *Ai14*. Detected fluorescence was quantified via high-content image analysis. Recombination was predominantly observed in the RPE and adjacent ciliary body. RPE flatmount preparations revealed a high level of recombination in adult mice (47.25–69.48%). Regional analysis of dorsal, ventral, nasal and temporal areas did not show significant changes in recombination. However, recombination was higher in the central RPE compared to the periphery. Higher levels of Cre-mediated recombinant expression was observed in embryonic RPE (~83%). Compared to other RPE-specific *Cre* transgenic mouse lines, this newly generated *RPE-Tyrosinase-CreEr*^*T2*^ line shows a more uniform and higher level of recombination with the advantage to initiate recombination in both, prenatal and postnatal animals. This line can serve as a valuable tool for researches exploring the role of individual gene functions, in both developing and differentiated RPE.

## Introduction

The retinal pigment epithelium (RPE) is a monolayer of pigmented epithelial cells intercalated between the neural retina and the choriocapillaris. With their long apical microvilli, RPE cells surround the light-sensitive outer segments of the retinal photoreceptors [1]. Due to its numerous functional roles, the RPE is essential for vision [2]. On the basal side, the RPE is in contact with the Bruch’s membrane and together they control ion, nutrient, water and metabolite transport between the retina and the retinal vascular network, the choriocapillaris [2,3]. Additionally, the RPE is important for re-isomerization of all-*trans*-retinol to 11-*cis*-retinal, phagocytosis of the shed photoreceptor outer segments, and absorption of incident light, all of which are essential for health and function of the photoreceptor cells [2–5]. RPE dysfunction and associated failure of one or more of these processes can lead to retinal degeneration and blindness [2]. Furthermore, dysfunction of the RPE has been associated with age-related macular degeneration (AMD), the most common cause of irreversible blindness in the elderly population [4]. In order to fully understand the molecular development and function of the RPE, and how dysregulation can affect visual function, RPE-specific gene knockouts are indispensable.

Homozygous germline knockout of widely expressed genes often leads to embryonic or neonatal lethality. Therefore, a tissue-specific gene-knockout strategy is required to determine tissue-specific effects of gene function. For this, the Cre/LoxP-system has emerged as the most commonly used method to introduce somatic mutations exclusively in the tissue of interest in mice [6,7]. In this system, a Cre recombinase is driven by a tissue-specific promoter and enables precise excision of a DNA sequence in the tissue of interest [4,5,7]. This technique enables either recombination-activated gene expression or conditional gene inactivation [6–8]. The site-specific Cre recombinase, derived from the P1 bacteriophage, is able to bind to a 34 bp recognition site (*loxP site*) and excise the DNA flanked between two *loxP* sites (floxed), leaving a single *loxP site* remaining [4,8–10]. Furthermore, conditional knockout of a gene in early development may have differing effects compared to ablation of gene function at a later time point [4,5,9]. Therefore, temporal gene expression also needs to be taken into account. To add a temporal dimension to the study of gene function, an inducible component can be added to the activation of the Cre [5,11]. The two most commonly used are the tetracycline-inducible Cre recombinase and the ligand-dependent 4-hydroxytamoxifen (4-OHT)-inducible Cre recombinase [4,7,11,12]. Due to its faster rate of induction, the 4-OHT-inducible Cre recombinase is more highly recommended [13]. Here, the Cre recombinase is fused to a ligand-binding domain (LBD) of the human estrogen receptor (ER) resulting in a CreER^T2^ construct, which is only activated upon addition of 4-OHT [6,11]. Taken together, a combination of a ligand-dependent tissue-specific promoter-driven Cre expression facilitates spatial and temporal control of Cre recombinase activity [7,11]. For this a mouse must inherit both, the gene for *Cre* and a floxed gene of interested, and be treated with tamoxifen, which will then be metabolized to its active form 4-OHT, at required time points [6,12].

Important considerations for choosing the *Cre* mouse lines are the level of expression in desired tissue of interest, and the level of ectopic expression. To date, few RPE-specific Cre mouse lines have been reported. Four non-inducible RPE-specific Cre lines include the *dopachrome tautomerase (Dct)-Cre* [8], the *tyrosinase related protein-1 (Tyrp1)-Cr*e [9], the *Melanoma-associated antigen recognized by T cells* (*MART1)-Cre* [14], and the *bestrophin-1 (BEST1)-Cre* [4]. All of these Cre recombinases lead to ectopic *Cre* recombination. The *Dct-Cre* line causes additional recombination in the telencephalon, the *MART1-Cre* line in the skin, and the *BEST1-Cre* line leads to ectopic *Cre* expression in the testis. However, in all, the expression in the eye is restricted to the RPE [4,8,14]. In contrast, the widely used *Tyrp1-Cre* line leads to ectopic *Cre* expression in the neuroretina, which might affect interpretations when studying retinal phenotypes caused by loss-of-function mutations in the RPE [9]. Thus far, two inducible RPE-specific *Cre* lines have been reported, the *monocarboxylate transporter 3 (Mct3)-Cre* [15] and the Tet-on *VMD2-Cre* [5]. Depending on the genetic background of the mice and the timing of induction, the *Mct-Cre* line only leads to low levels of recombination (5–20%) [4,15]. For the *VMD2-Cre* line, the level of recombination has not been reported. However, at P4 it shows the greatest activity [4,5], suggesting that it might be less efficient for manipulation of genes expressed at earlier time points.

To address the above shortcomings, we generated an inducible RPE-specific transgenic mouse line with uniform recombination efficiency. Since the 4-OHT-inducible CreEr^T2^ is considered the most efficient driver for inducible genetic recombination [6], we generated a transgenic mouse line using this Cre recombinase under transcriptional control of a previously published and validated RPE-specific enhancer of the *Tyrosinase* gene [16,17]. *Tyrosinase* (*Tyr*) is expressed in all pigmented cells. However, it is differentially regulated between the RPE and melanocytes due to a novel tissue-specific distal regulatory element (*Cns-2*), which is located at -47 kb from the transcription start site [17]. *Cns-2* is responsible for *Tyrosinase* gene expression in the RPE, but not in melanocytes. In this study, we characterize our inducible RPE-specific Cre mouse line and evaluate its level of recombination efficiency by crossing it with a commonly used reporter mouse line.

## Material and methods

### Generation of a transgenic RPE-Tyrosinase-CreEr^T2^ mouse line

A *RPE-Tyrosinase-CreEr*^*T2*^ transgenic mouse line (strain *C57BL/6N*) was generated by insertion of a (5’-3’) construct containing a RPE-specific *Tyr enhancer* (*Cns-2*) (4721 bp) [17], a *hsp70 minimal promoter* (985 bp) and a construct for the inducible Cre-ER^T2^ recombinase [11] (1983 bp). *Cre-Er*^*T2*^ was amplified from the pCAG-CreERT2 vector (Addgene) and cloned into the Tyr-Hsp70 vector using XhoI/SalI enzymes. Generation of the *Tyr-GFP* mouse line was previously reported [16].

### Animals

All experiments had ethical approval from the Landesuntersuchungsamt Rheinland-Pfalz and were performed in accordance with the guidelines given by ARVO Statement for the Use of Animals in Ophthalmic and Vision Research. Animal maintenance and handling were performed in line with Federation for Laboratory Animal Science Associations (FELASA) recommendations. Mice were housed in a 12 h light/dark cycle. Animals were sacrificed by cervical dislocation. The morning after mating was considered as E0.5. From the time at which the mice were six weeks old, they were considered adult.

*B6*.*Cg-Gt(ROSA)26Sor*^*tm14(CAG-tdTomato)Hze*^*/J* reporter mice (JAX stock #007914; [18]; referred to as *Ai14*) were crossed with *RPE-Tyrosinase-CreEr*^*T2*^ mice to visualize *Cre* expression. *Ai14* mice possess a floxed stop cassette upstream of the CAG promoter-driven red fluorescent protein tdTomato. Following Cre-mediated recombination the stop cassette is excised, allowing expression of tdTomato.

### Genotyping

For DNA isolation the tissue samples were incubated in tissue digestion buffer (10 mM Tris-HCl pH 8.0, 10 mM EDTA, 200 mM NaCl, 0.5% SDS, 0.1 mg/ml Proteinase K) overnight at 55 °C. Afterwards the samples are vortexed and centrifuged at 21,130 rcf for 10 min. The supernatant was transferred carefully into a new tube and incubated with double the volume of 95% Ethanol for at least 10 min at room temperature. After centrifugation at 21,130 rcf for 15 min, the DNA pellet was air dried at room temperature and dissolved in Milli-Q water to a concentration between 100 and 200 μg/ml. PCR of genomic DNA was performed using GoTaq G2 Hot Start Polymerase (Promega). For detection of the *Cre* construct, the following primers were used Cre-F (5'-GAGTGAACGAACCTGGTCGAAATCAGTGCG-3') and Cre-R (5'-GCATTACCGGTCGATGCAACGAGTGATGAG-3'). To detect a ~300-bp product using the following cycling conditions: 90 s at 94 °C for initial denaturation, followed by 40 cycles of 30 s at 94 °C for denaturation, 30 s at 55 °C for annealing, 60 s at 72 °C for extension, and finally 5 min at 72 °C for final extension. To detect the *Ai14* construct, the Jackson Laboratory (www.jax.org) provided primer sequences and thermocycling conditions. All PCR products were separated on a 2% agarose gel and visualized using GelRed Nucleic Acid Gel Stain.

### Cre recombinase activity in transgenic mice

To induce Cre recombination activity in the mice, 1.6 mg Tamoxifen (Sigma-Aldrich, T5648) and 1.6 μg β-Estradiol (Sigma-Aldrich, E8875) were administered on five consecutive days via intraperitoneal injection (IP) or oral gauvage (OG). Tamoxifen was prepared as a 10 mg/ml stock solution in flax seed oil. The solution was incubated at 37 °C and vortexed occasionally until dissolved. β-Estradiol was added to the tamoxifen solution at a concentration of 10 mg/ml. Treatment of pregnant dams was started at E9.5 and embryos were harvested at E17.5. For treatment in adults, eyes were harvested on the fifth day after the last application. For short-term (ST) analyses of Cre activity eyes were harvested five days after the last treatment application. For long-term (LT) Cre activity eyes were harvested three months after the last treatment application.

### Quantitative real-time PCR (qRT-PCR)

Tissues of adult mice were harvested on the fifth day (ST) or three months (LT) after the last application and homogenized in TRIzol Reagent (Invitrogen) using a pestle. For RNA extraction, the TRIzol Reagent was used according to manufacturer’s recommendations. RNA was stored at -80 °C. RNA was reverse transcribed to cDNA using GoTaq Probe 2-Step RT-qPCR System (Promega) and cDNA was stored at -20 °C. qRT-PCR was performed via a StepOnePlus Real-Time PCR System (Applied Biosystems) using Platinum SYBR Green (Invitrogen). The following cycling conditions were used: 95 °C for 10 min followed by 40 cycles of 95 °C for 15 sec, 60 °C for 1 min. Relative target gene expression was normalized to *TBP*. Primer sequences are listed in Table 1.

**Table 1:** Primers used for qRT-PCR.

Gene	Species	Forward	Reverse
*Lrat*	Mouse	TTCAAGCTCTTTAGCGTGAGC	TTTCATAGGGACGGTTCTTCC
*Rpe65*	Mouse	TTGAAACTGTGGAGGAACTGTC	GACTGCCAGTGAGCCAGAG
*Ttr*	Mouse	CCTCGCTGGACTGGTATTTG	GACCATCAGAGGACATTTGG
*Tbp*	Mouse	CTTCGTGCAAGAAATGCTGAAT	CAGTTGTCCGTGGCTCTCTTATT

### Fluorescence staining and mounting of flatmount RPE and retina

For RPE or retina staining, mice were sacrificed, eyes were enucleated and the retina was removed. The tissue was fixed with 4% paraformaldehyde (PFA) in 1× phosphate-buffered saline (PBS) for 1 h and washed three times with 1× PBS. To reduce auto-fluorescence from the PFA, the tissue was incubated with 50 mM NH_4_Cl for 10 min before permeabilizing with 1× PBS 0.1% Tween-20 (PBST) with 0.3% Triton-X (TX) (PBST-TX) for 1 h and blocked with blocking buffer (0.1% ovalbumin, 0.5% fish gelatin in 1× PBS) over night at 4 °C. Eyecups were incubated with DAPI (Carl Roth) and a directly conjugated Zonula Occludens-1 (ZO-1) antibody, conjugated to Alexa Fluor 488 (ZO-1-1A12, Invitrogen, 339188) in blocking buffer for 2 h in dark conditions at room temperature. Post staining, two washing steps with PBST-TX and one with 1× PBS for 20 min each were performed. Eyecups or retina were flat mounted with Fluoromount-G (SouthernBiotech) and examined using a fluorescence microscope (ZEISS Axio Scan.Z1, Leica DM6000 B). Entire optic cup imaging was performed at either 10× or 20× magnification across three channels (red: tdTomato, green: ZO-1, and blue: DAPI). Exposure times were kept consistent at each magnification with scaling of the exposure time between magnifications to adjust for the decreased numerical aperture of the 10× objective.

### Fluorescent staining and transmission electron microscopy of eye and embryo sections

For RPE or retina staining, mice were sacrificed, eyes were enucleated and immediately fixed with 4% paraformaldehyde (PFA) in 1× phosphate-buffered saline (PBS). After 5 min of fixation a hole was poked through the middle of the cornea and the eyes were incubated again in 4% PFA for another 15 min. Following this, the eyes were cut along the ora serrata and the anterior segment was removed, leaving the lens intact. The resulting eyes were fixed in 4% PFA for another hour. Post fixation, four washing steps with 1× PBS for 15 min each were performed. Afterwards the eyes were incubated in following sucrose solutions, each made with 1× PBS: once in 10% sucrose solution for 30 min, twice in 20% sucrose solution for 20 min each and once in 30% sucrose solution overnight. The next day the eyes were again incubated in 30% sucrose solution for 1 h. Afterwards the eyes were cryofixed in isopentane, cryosectioned and immunostained as previously described [19]. For immunostaining, the sections were placed on poly-L-lysine coverslips and permeabilized with 0.5% PBS-TX for 10 min before incubation with 50 mM NH_4_Cl for 10 min. After a washing step with 1× PBS for 5 min, the sections were blocked with blocking buffer for 30 min at room temperature and then incubated with monoclonal primary antibodies against ADP-ribosylation factor-like protein 13B (Arl13b) (Sigma-Aldrich), Calbindin (Millipore), Calretinin (Millipore), Protein kinase C α (PKC α) (Sigma) or Glutamine synthetase (GS) (Abcam), diluted in blocking buffer, overnight at 4 °C. Three washing steps with 1× PBS, 10 min each, were performed before incubation with secondary antibodies conjugated to Alexa-488 (Invitrogen) and DAPI, diluted in blocking buffer, for 2 h in dark conditions at room temperature. Post staining, three washing steps with 1× PBS and one with Milli-Q water, 10 min each, were performed and the sections were mounted using Mowiol 4–88. The sections were examined using a fluorescence microscope (Leica DM6000 B) and the images were processed via FIJI using color correction and contrast adjustment. Whole E16.5 embryos were fixed in 4% paraformaldehyde overnight at 4 °C, then washed in 1× PBS and transferred to 30% sucrose/1× PBS until the embryos sank. Embryos were then incubated in a 1:1 mixture of OCT and 30% sucrose/1× PBS for 30 min on a rocking platform. After transferring embryos to an embedding mold containing OCT, they were oriented as desired and frozen on dry ice. Frozen blocks were cut on a microtome, stained with DAPI and imaged using fluorescence microscope (ZEISS Axio Scan.Z1). For transmission electron microscopy (TEM), dissected eyecups were fixed in 2.5% glutaraldehyde in 0.1M cacodylate buffer with 0.1M sucrose for 1 h at room temperature. Post fixation they were placed in 2% osmium tetroxide (in 0.1M cacodylate buffer) for 1 h and then dehydrated through an ethanol series. After embedding in epoxy resin, eyes where cut and processed for TEM using standard EM procedures.

### Image analysis

Images were processed using a custom FIJI plugin. Areas of expression in all regions were assessed for all cells across all 15 eyes. Areas of coverage were then aggregated across Male IP, Female IP, and Female OG and a statistical analysis was performed to determine if expression was heterogeneous. Whole optic cup images were separated into their respective channels. Background subtraction and intensity scaling was performed in each channel so that 99% of the signal obtained in the image fell within the dynamic range of the image (16-bit image). Background subtraction and scaling was done to transform pixel intensity histograms between all images into similar distributions. After scaling, images were converted to 8-bit images. Images whose initial intensity distribution took less than 5% of the dynamic range of the image were removed from analysis due to the lack of signal to noise. Images were then duplicated into a “Foreground” image and a “Background” image. The Foreground images were used to quantify expression levels and thus were not further edited. The Background images were used to identify areas in the images that could not be measured (bubbles or tissue damage) or that did not contain tissue (background). No expression quantification was done on Background images.

In Background images, all pixels first had one intensity unit added to them. Then, areas in images that contained artifacts (bubbles or damaged tissue) or no tissue were manually removed (given a 0-value). After giving a 0-value to all non-measurable/background areas in an image a background mask was created in which the location of all 0-value pixels was identified. This mask was overlaid onto the Foreground image and all background pixels were given a 0-value in the Foreground Image. The total area of the background pixels was subtracted from the total image area to identify the total possible area of expression (*A*_*Tot*_). All Foreground images were then segmented using a manually validated threshold. The area segmented as expressing (*A*_*Exp*_) was then divided by *A*_*Tot*_ to determine the total percent of expression (A_*Per*_). Representative image processing is shown in supplementary information (S5 Fig).

$$\frac{A_{Exp}}{A_{Tot}}=A_{Per}$$ Eq 1

In adult mice, nasal, temporal, dorsal, ventral, central, and peripheral areas where then manually cropped in each image and Eq 1 was used to determine the percent expressing in each cropped image. The central region was determined as an ellipsoid region that was half the diameter of the total butterflied eyecup in each orthogonal direction (nasal/temporal and dorsal/ventral). All area outside this region was defined as the periphery.

In fetal eyes, Cre activity was predominately found in the nucleus and little tdTomato expression after Cre mediated recombination was seen in the cytoplasm. Thus, expression in these eyes was performed using two methodologies: (1) by assessing the total area of nuclear expression and total area of Cre/tdTomato expression and dividing the respective areas as shown in Eq 1. (2). Across seven fetal eyes 73,499 cell nuclei were measured. To ensure the robustness of this method the total count of nuclei (N_Nuc_) was also determined as well as the total count of nuclei that expressed Cre/tdTomato (N_Exp_). The ratio of these was then taken to determine the total percent expressing (N_Per_). Eq 2 shows this formula below.

$$\frac{N_{Exp}}{N_{Nuc}}=N_{Per}$$ Eq 2

### Statistics

All comparisons between treatments were performed with a linear mixed effect (LME) model controlling for the repeated measures performed on each eye (Total expression and regional expression) as well as for multiple eyes coming from each mouse (2 eyes per mouse). An LME model was used because several eyes were removed due to lack of staining (intensity histogram represented less than 5% of the dynamic range of the image) and thus modeling approaches that could allow for “missing” data were necessary. Multiple comparisons were controlled for using Tukey’s Post-Test. Normality was tested for using the Kolmogorov-Smirnov (KS) normality test. All statistical analysis was performed using R [20] with the “nlme” package [21] and the “multcomp” package [22]. Significance was set as less than 0.05.

## Results

### Generation of *Cre* transgenic mice

**Fig 1:**
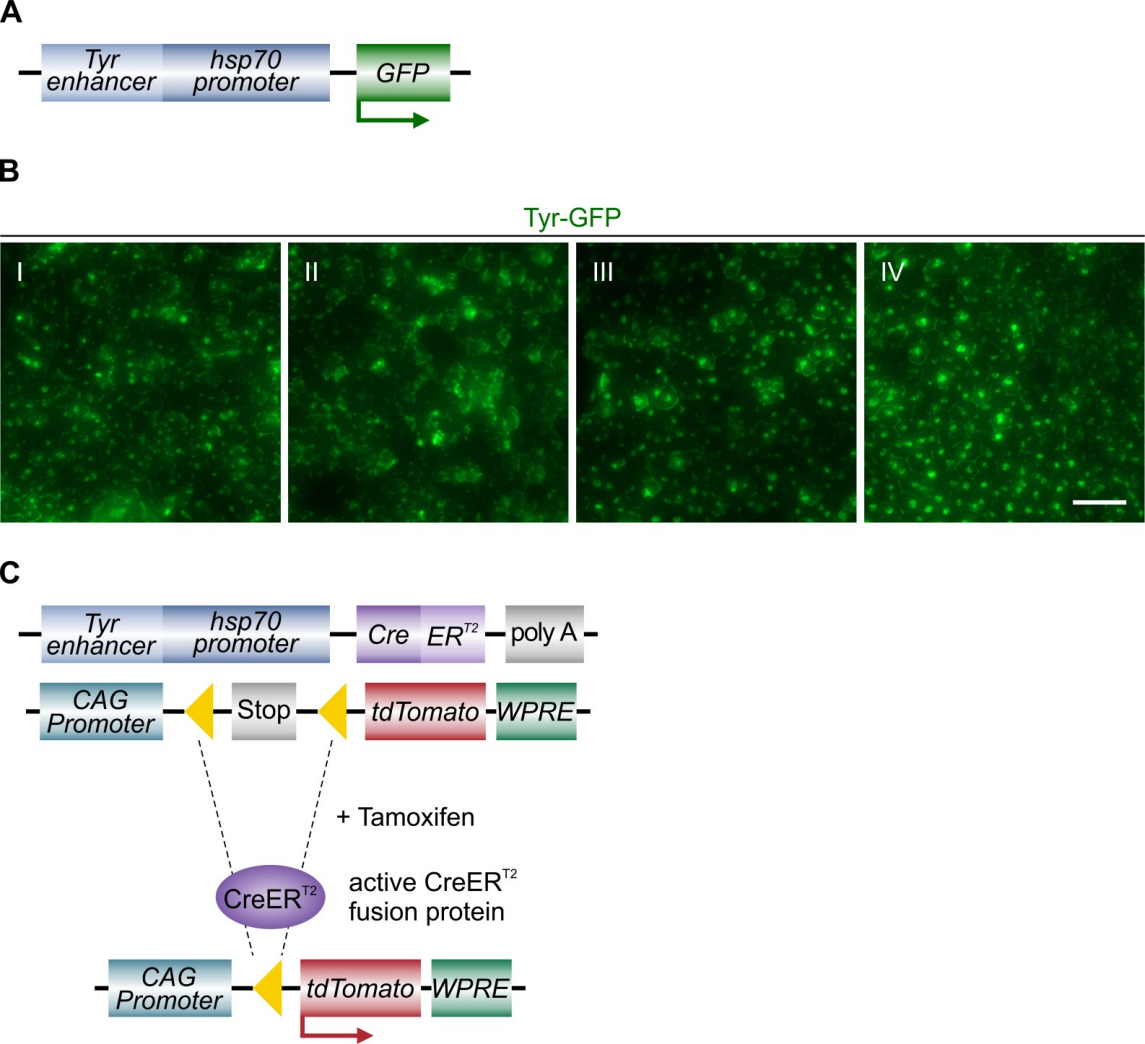
Generation of tamoxifen-inducible Cre transgenic mice. A: Schematic of the RPE-specific *Tyr-GFP* construct used for generation of *Tyr-GFP* mice. B: Regional examples (I-IV) of *Tyr-GFP* expression showing comprehensive expression of GFP under control of the *Tyr* promoter. Images taken from the flatmount shown in S1 Fig. C: Schematic of the induction of Cre activity in double transgenic mice. Mice harboring the *RPE-Tyrosinase-CreEr*^*T2*^ cassette and the *Ai14* cassette are treated with tamoxifen to induce Cre activity. Following recombination, the floxed exons (yellow triangles) are excised. In the case of the *Ai14* cassette, recombination removes a stop sequence allowing for expression of tdTomato. Scale bar: (B) 50 μm.

To generate an RPE-specific *Cre* transgene, a *hsp70* promoter was placed downstream of a RPE-specific *Tyr* enhancer *Cns-2* [17], as previously described [23]. To verify the expression of this combined construct containing both, the *Tyr* enhancer *Cns-2* and *hsp70* promoter, a *GFP* cassette was fused downstream (*Tyr-GFP*) (Fig 1A) [16]. As previously observed in embryonic tissue, this *Tyr-GFP* construct was also spatially restricted to the RPE in the adult eye. We observed GFP expression distributed throughout the whole RPE in adult mice (Fig 1B, S1 Fig). To obtain a tamoxifen-inducible *RPE-Tyrosinase-CreEr*^*T2*^ transgene, the *GFP cassette* was replaced by a *CreER*^*T2*^ cassette [11] (Fig 1C). In order to characterize the specificity and efficiency of this inducible RPE specific *Cre* recombinase, we crossed the *RPE-Tyrosinase-CreEr*^*T2*^ mouse line with a *tdTomato* reporter line, namely *Ai14*. This mouse line contains a floxed *Stop* cassette upstream of the gene for the red fluorescent protein *tdTomato* [18], and is therefore able to express *tdTomato* after tamoxifen treatment (Fig 1C).

### Analysis of Cre-mediated recombination in *Ai14;RPE-Tyrosinase-CreER*^*T2*^ mice

**Fig 2:**
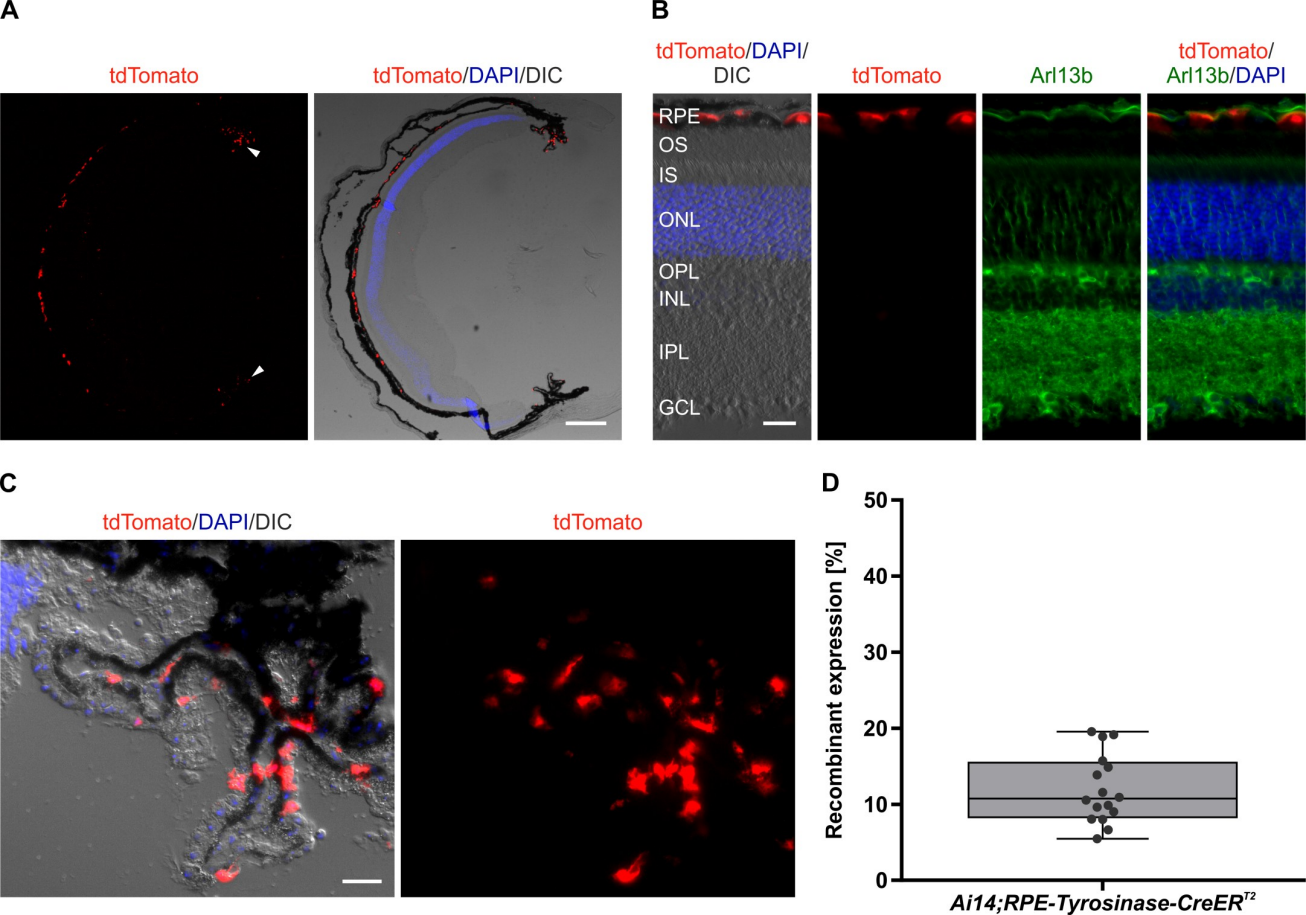
Cre-mediated recombinant expression in the RPE of adult *Ai14;RPE-Tyrosinase-CreEr*^*T2*^ mice. A, B: Localization of tdTomato expression in an adult eye upon Cre activity in longitudinal cryosections stained with DAPI (blue) and Arl13b (green) to visualize the connecting cilium (B), respectively. Differential interference contrast (DIC) image was overlaid with the DAPI staining and red fluorescent tdTomato expression. Cre activity resulted in tdTomato expression, which is seen in the RPE and to a lesser extent in the ciliary body (white arrowheads). C: Cre activity resulted in simultaneous tdTomato expression in the ciliary body. D: Quantification of tdTomato expression in the ciliary body. n = 16 ciliary bodies from three individual animals. RPE: Retinal pigment epithelium, OS: Outer segments, IS: Inner segments, ONL: Outer nuclear layer, OPL: Outer plexiform layer, INL: Inner nuclear layer, IPL: Inner plexiform layer, GCL: Ganglion cell layer. Scale bars: (A) 250 μm, (B) 25 μm, (C) 25 μm.

**Fig 3:**
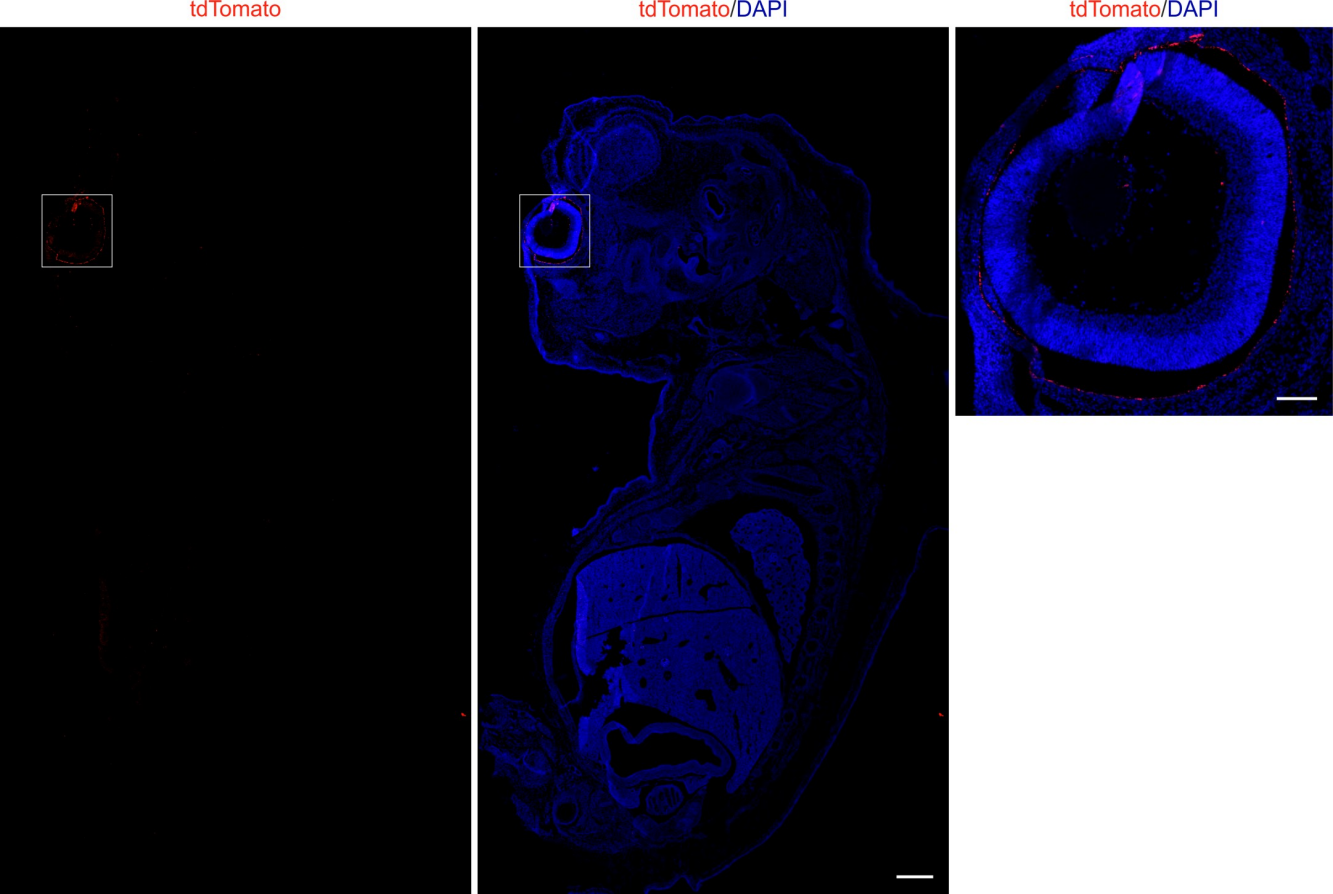
Cre-mediated recombinant expression in an *Ai14;RPE-Tyrosinase-CreEr*^*T2*^ embryo section. A: Fluorescent image of tdTomato expression in longitudinal embryo sections stained with DAPI (blue) showing that Cre activity is restricted to the eye. B: Higher magnification of the eye region in A shows high specificity for the RPE. Scale bar: (A) 500 μm, (B) 100 μm.

**Fig 4:**
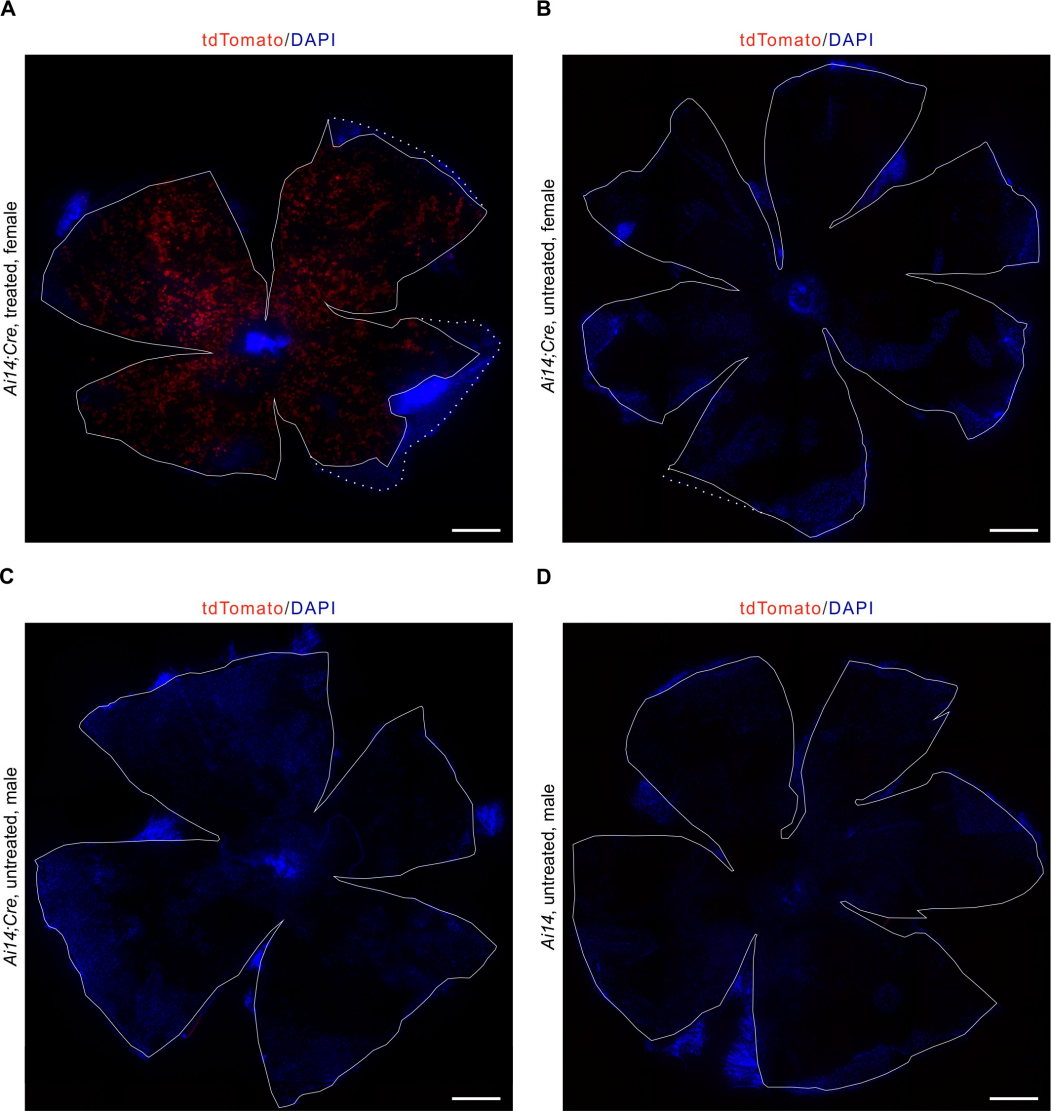
Representative RPE flatmount preparations from control animals. A: RPE flatmount of a treated female adult *Ai14;RPE-Tyrosinase-CreEr*^*T2*^ mouse showing tdTomato expression throughout the whole RPE. B: RPE flatmount of an untreated female adult *Ai14;RPE-Tyrosinase-CreEr*^*T2*^ mouse showing no red fluorescence, thus tdTomato expression. C: RPE flatmount of an untreated male adult *Ai14;RPE-Tyrosinase-CreEr*^*T2*^ mouse showing no red fluorescence, thus tdTomato expression. D: RPE flatmount of an untreated male adult *Ai14* mouse, being *Cre* negative. No red fluorescence, thus tdTomato expression, was observed. All flatmounts were counterstained with DAPI (blue). Scale bar: (A-D) 500 μm.

To evaluate recombinant expression of our generated transgene, we analyzed the expression of tdTomato in *Ai14;RPE-Tyrosinase-CreEr*^*T2*^ mice RPE. After five days of consecutive tamoxifen treatment, the mouse eyes were harvested on the fifth day after the last dose, and prepared for flatmount preparations or cryosections. Recombinant expression of tdTomato was detected via fluorescence microscopy (Fig 2). In longitudinal cross-sections, we predominantly observed recombination in the RPE (Fig 2A and 2B, S3 Fig) as well as in the ciliary body (Fig 2A, white arrowheads). Quantification showed relatively low recombinant expression in the ciliary body (~10.71%) (Fig 3C and 3D). In contrast, retina flatmounts from treated animals showed virtually no tdTomato expression (Panel A in S2 Fig). Longitudinal cross-sections revealed occasionally weak ectopic expression in the inner plexiform layer (IPL) of the retina (S3 Fig). To identify any possible cell type specificity of the occasional ectopic expression in the inner retina, we performed immunohistochemistry with a variety of inner retina markers (Calbindin, Calretinin, Protein kinase C alpha (PKC α), Glutamine synthetase (GS)). In the rare event of red fluorescence in the retina, we never found any co-expression with these inner retina markers, suggesting that ectopic expression was not observed in horizontal cells, amacrine cells, rod bipolar cells or Müller glia cells. Therefore, this expression is not in any of the dominant retinal cell types. To confirm that tdTomato expression was not detected in any other tissue types outside of the eye, we prepared whole mouse embryo sections at E16.5. Whole body imaging confirmed that recombination was only restricted to the eye (Fig 3A), and higher magnification of the eye also showed specificity for the RPE and absence of recombination in the retina (Fig 3B). To test for leakiness of Cre expression, we generated flatmount preparations from a variety of control mice, and looked for any unspecific activation of Cre (Fig 4). In contrast to a treated *Ai14;RPE-Tyrosinase-CreEr*^*T2*^ mouse, we never detected any Cre activity in untreated *Ai14;RPE-Tyrosinase-CreEr*^*T2*^ mice. We confirmed this in both male and female mice. To exclude any preparation artifact that might lead to fluorescent detection, we also prepared a RPE flatmount from an untreated *Ai14* mouse that did not contain the *RPE-Tyrosinase-CreEr*^*T2*^ construct. No fluorescent signal could ever be detected. Since application of β-Estradiol assists late stages of gestation and initiation of pup delivery, [24], we added β-Estradiol to our treatment solutions in both pregnant (see below) and non-pregnant mice. To exclude unintended activation of the Cre recombinase via addition of β-Estradiol, we treated animals with β-Estradiol alone, dissolved in flax seed oil at the same concentration as in the tamoxifen solution (Panel B in S2 Fig). Our data showed that β-Estradiol alone was not able to drive recombination via Cre-ER^T2^.

### Efficacy and assessment of homogeneity of Cre-mediated recombination in adult mice

**Fig 5:**
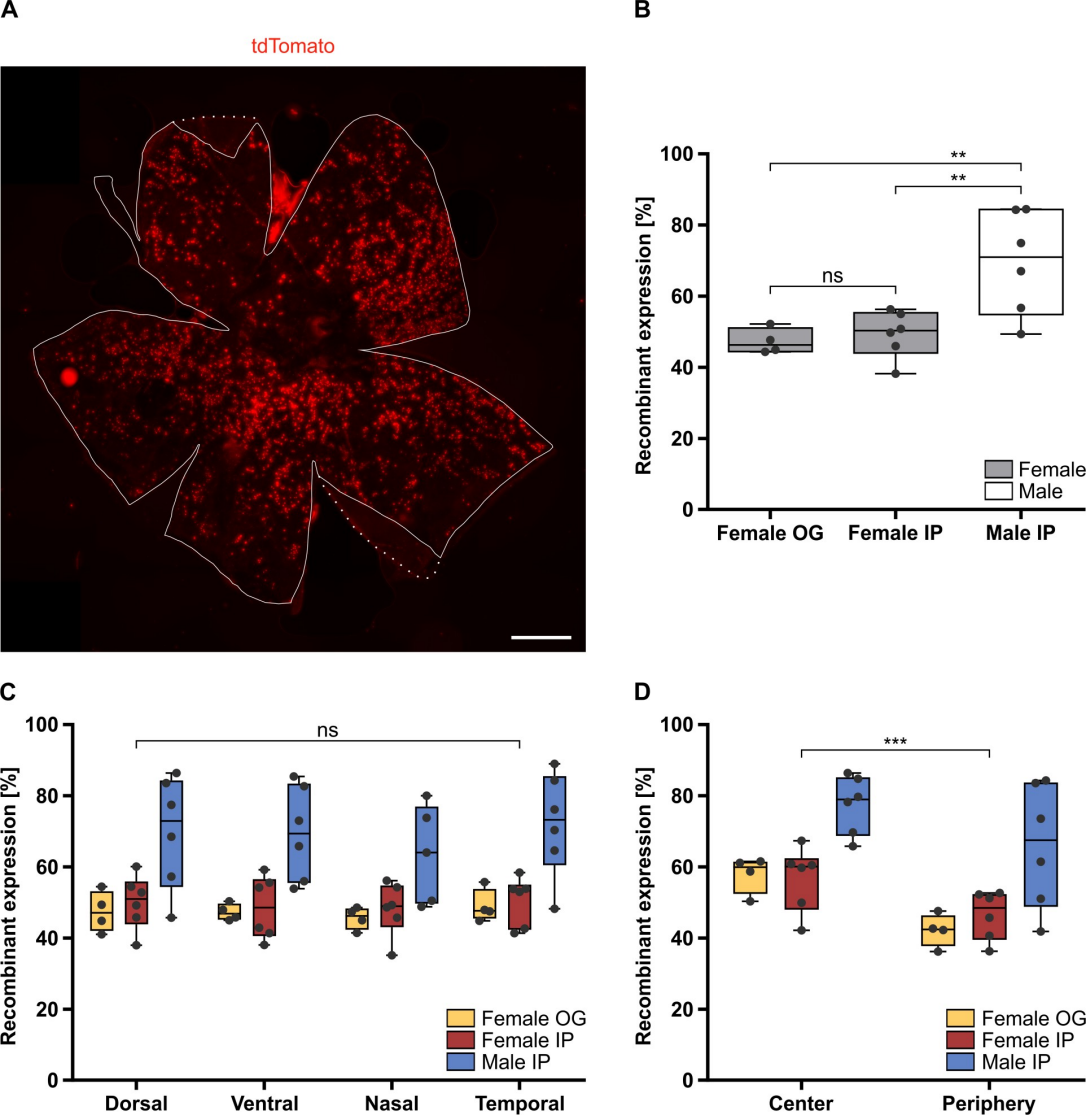
Analysis of Cre-mediated recombinant expression and its overall distribution in the adult *Ai14;RPE-Tyrosinase-CreEr*^*T2*^ RPE. A: Fluorescent image showing the uniform distribution of tdTomato expressing cells throughout an adult RPE flatmount preparation. Outline of the RPE indicated by the solid line, dotted line represents inverted choroidal tissue. B: Quantification of recombinant expression in RPE of female mice, treated via oral gavage (OG) (47.25%) (n = 4 eyes) compared to female mice treated via intraperitoneal injection (IP) (49.35%) (n = 6 eyes) showed similar levels of recombinant expression (p = 0.949). The difference between male (69.48%) (n = 6 eyes) and female mice, both treated via IP, is statistically significant (p = 0.0029). C: Quantification of the recombinant expression in dorsal, ventral, nasal and temporal areas of the RPE in three different treatment conditions (Female OG, Female IP and Male IP) showed no significant regional differences (p = 0.9995, p = 0.5315). D: Quantification of the recombinant expression in central vs. peripheral areas of the RPE in the three different treatment conditions (Female OG, Female IP and Male IP) showed a significant difference (p = 8.60E-07). Significance levels: > 0.05 not significant (ns), ≤ 0.05 *, ≤ 0.01 **, ≤ 0.001 ***. Scale bar: (A) 500 μm.

To quantify the efficiency of the recombinant expression of our generated transgene, we analyzed the expression of tdTomato via software analysis in *Ai14;RPE-Tyrosinase-CreEr*^*T2*^ mice. Mice were treated as mentioned above and recombinant expression of tdTomato was detected via fluorescence microscopy. Flatmounts of the RPE showed a uniform distribution of tdTomato expression throughout the entire RPE (Fig 5A). Depending on gender, we observed between 47.25% and 69.48% recombinant expression in adult mice (Fig 5B). In female mice, application via oral gavage (OG) did not show any significant differences compared to intraperitoneal injection (IP) (p = 0.949). Whereas, IP injections was significantly more efficient in male mice compared to female mice (p = 0.0029).

Additionally, we quantified the homogeneity of recombinant expression across the entire RPE monolayer. In order to determine any spatial differences in the level of tdTomato expression and recombination, we divided the RPE flatmounts into dorsal, ventral, nasal and temporal regions (Panel A in S5 Fig) and quantified the level of tdTomato via high-content image analysis (Fig 5C). We saw no significant differences between nasal-temporal and dorsal-ventral expression in any of the treatment conditions tested, thereby confirming that that our newly generated *RPE-Tyrosinase-CreEr*^*T2*^ driver can be used to target the entire RPE in adult animals. Similarly, we analyzed the distribution of tdTomato expression in center versus peripheral areas of the RPE flatmounts (Panel B in S5 Fig). The center was defined on the RPE flatmount at the optical nerve. As seen in Fig 5D, there was only a mean difference of 12% expression between center (64.80%) and periphery (52.71%; p = 8.60E-07) across all treatment groups despite the area ratio between center and periphery being 25%.

### Efficacy of Cre-mediated recombination in embryonic mice

**Fig 6:**
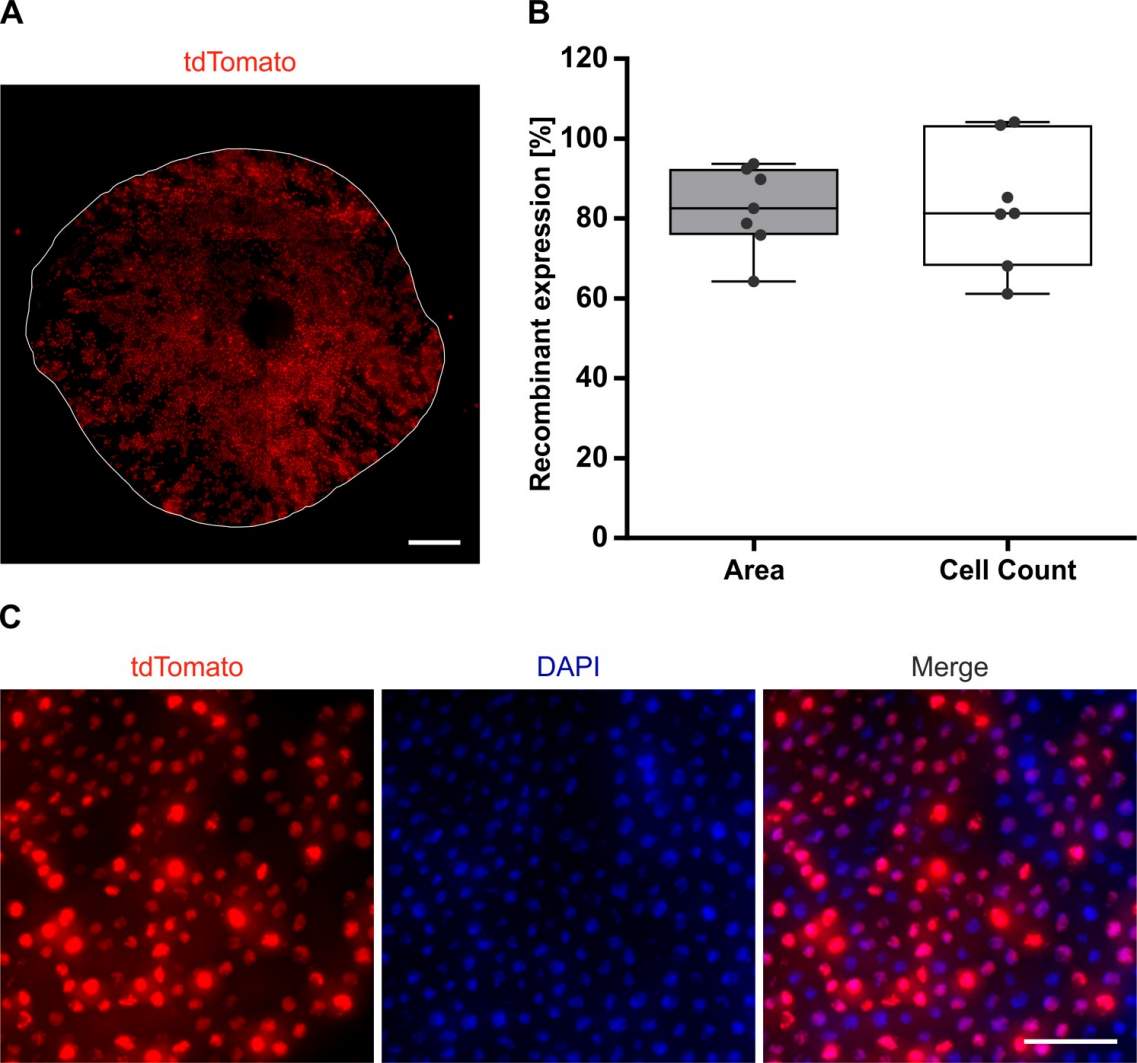
Analysis of the Cre-mediated recombinant expression in embryonic *Ai14;RPE-Tyrosinase-CreEr*^*T2*^ mice. A: Fluorescent flatmount image showing the uniform distribution of tdTomato expressing cells throughout the RPE of an E17.5 *Ai14;RPE-Tyrosinase-CreEr*^*T2*^ embryo. Outline of the RPE indicated by the solid line. B: Upon quantification method, recombinant expression in embryonic mice was 82.5% (Area) and 83.5% (Cell Count), respectively (n = 7 eyes). C: Representative fluorescent flatmount images showing tdTomato expression and DAPI staining of an E17.5 *Ai14;RPE-Tyrosinase-CreEr*^*T2*^ RPE. Scale bar: (A) 200 μm, (C) 50 μm.

Since many developmental studies address the role of gene expression during RPE development, we wanted to determine the level of *Cre* recombination driven by our *RPE-Tyrosinase-CreEr*^*T2*^ construct achieved in embryonic tissue. For this, timed pregnant *Ai14;RPE-Tyrosinase-CreEr*^*T2*^ mice were treated with tamoxifen in order to trigger Cre-mediated recombination embryonically. Since *Tyr* expression starts at E10.5 [17], we began treatment at E9.5. Following five days of treatment, embryos were collected at E17.5, the RPE was dissected for flatmount preparations, stained and mounted for analysis of recombinant expression of tdTomato via fluorescence microscopy (Fig 6). RPE flatmounts showed a uniform distribution of tdTomato throughout the whole RPE monolayer (Fig 6A). Two separate methods for quantification (described in the methods) revealed similar levels of recombinant expression, namely 82.50% (Area) vs. 83.51% (Cell Count) (Fig 6B). For the Cell Count method, two values over 100% were obtained. This was caused by detection of more tdTomato positive cells than DAPI positive cells. Representative higher magnified images of a RPE flatmount are shown in Fig 6C. Manual counting of this smaller region revealed 77.87% recombinant expression (Nuclei: 235, tdTomato: 183). Manual counting of additional regions from different RPE flatmounts resulted in 82.35% recombinant expression (82.35% ± 2.97% SD, 2735 nuclei counted), suggesting that our automated software was reliable. These results demonstrate that our newly generated *Tyr-CreER*^*T2*^ mouse line can also be employed as a tool for developmental studies.

### Analysis of toxicity after Cre activation

**Fig 7:**
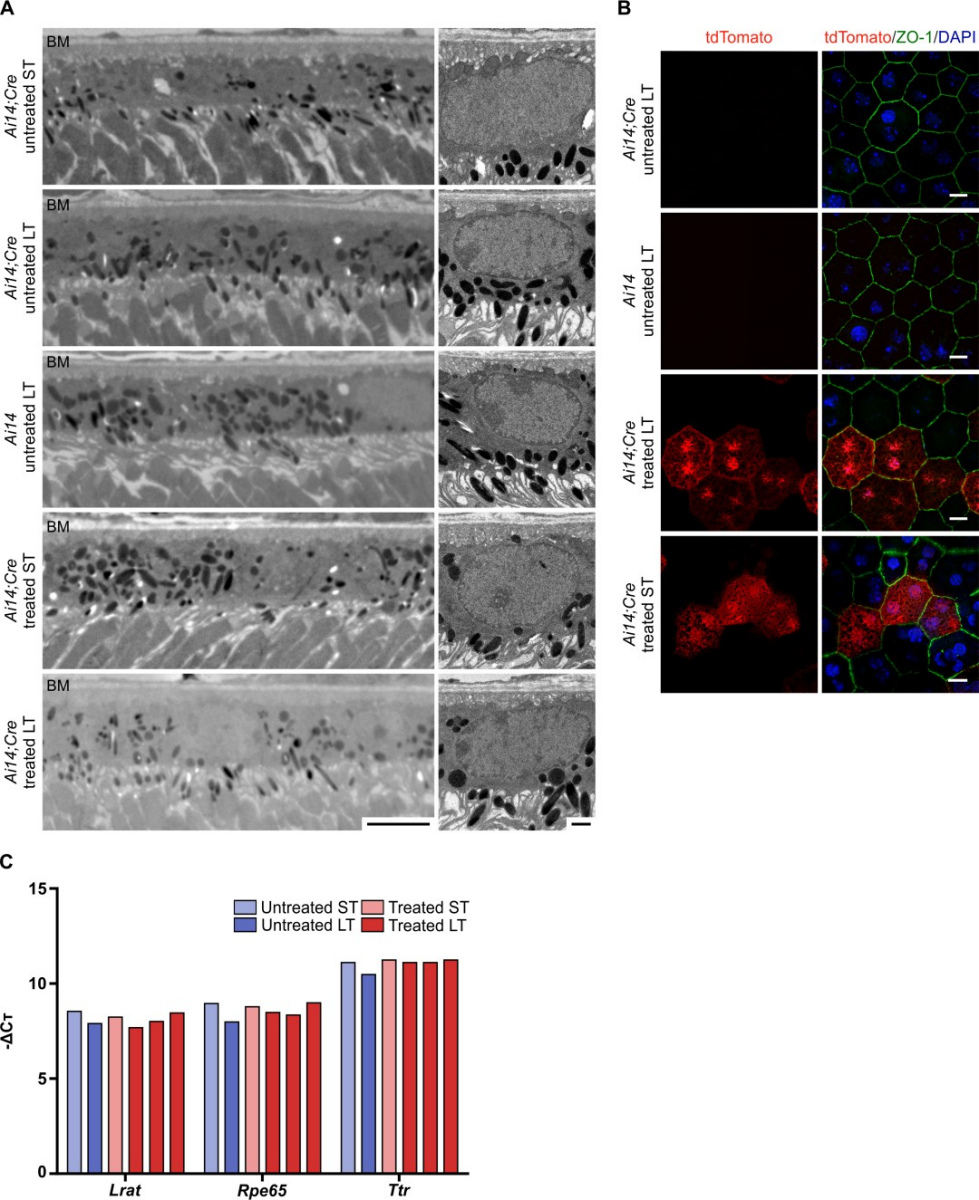
Analysis of the long-term effect of Cre activity on RPE cells of adult *Ai14;RPE-Tyrosinase-CreEr*^*T2*^ mice. A: Electron micrographs of adult RPE at lower (left) and higher magnification (right), showing no phonotypical abnormalities after short- and long-term Cre activity. B: Flatmount preparations of adult RPE after short-term and long-term Cre activity, showing no morphological changes in cells expressing tdTomato expression (red) and no expression in untreated mice. The tight junction-associated protein ZO-1 (green) was labeled to visualize the cell borders. C: qRT-PCR shows no difference in expression of mature RPE markers (*Lrat*, *Rpe65* and *Ttr*) relative to housekeeping gene (*Tbp*). *Ai14;Cre*: *Ai14;RPE-Tyrosinase-CreEr*^*T2*^. ST: short-term effect of tamoxifen-treatment. LT: long-term effect of tamoxifen-treatment. BM: Bruch’s membrane. Scale bars: (A) right: 5 μm, left: 1 μm, (B) 10 μm.

Since Cre expression can sometimes lead to cellular toxicity, we checked for pathological phenotypes in the RPE of treated mice [12]. High-resolution histological preparations, imaged via transmission electron microscopy (TEM), showed no morphological changes of the RPE and surrounding structures (Bruch’s Membrane), either after short-term (up to five days) or long-term (up to three months) Cre activity (Fig 7A). Higher magnification of fluorescently labeled RPE flatmounts, also showed no morphological changes in tdTomato expressing cells, based on cellular morphology and epithelial patterning, either after short-term or long-term Cre activity (Fig 7B). Again, suggesting that Cre expression is not detrimental to cellular homeostasis. Gene expression of mature RPE markers (*Lecithin retinol acyltransferase* (*Lrat*), *Retinal pigment epithelium-specific 65 kDa protein* (*Rpe65*), *Transthyretin* (Ttr)), as measured by quantitative real-time PCR (qRT-PCR), showed no differential expression between treated and untreated mice of the same age (Fig 7C). Furthermore, no difference in gene expression was observed between RPE tissues exposed to short- vs. long-term Cre activity.

## Discussion

With its numerous functions, the RPE is unconditionally essential for the health and function of the retina and thereby for visual processes. Since RPE dysfunction is associated with retinal degeneration and blindness [2], it is essential to understand molecular development and function of the RPE. Currently available RPE Cre recombinases show lack of specificity or poor recombination. Therefore, we sought to generate an alternative. Here we show that our *RPE-Tyrosinase-CreEr*^*T2*^ transgenic mouse line will be a valuable tool for spatially and temporally controlled Cre activation in both, embryonic and adult mouse RPE.

Crossing our *RPE-Tyrosinase-CreEr*^*T2*^ transgenic mouse line with a reporter mouse line (*Ai14*) enabled us to evaluate the recombination efficiency and specificity. Embryo sections revealed a specificity for ocular tissues. As shown in the immunostained eye sections, activity of the *RPE-Tyrosinase-CreEr*^*T2*^ is mainly restricted to the RPE. Minimal ectopic expression was detected in the INL of the retina, where no cell type-specificity was observed. Also retina flatmounts revealed that this level of ectopic expression is negligible. Additionally, relatively low expression was also observed in the pigmented cells of the ciliary body. Since the RPE extends into the pigmented parts of the ciliary body and iris during development [25], this finding was not unexpected. Importantly, analysis of the recombinant expression of tdTomato in the RPE revealed that Cre recombinase activity did not lead to any morphological changes in cell morphology, proving that Cre recombinase activity did not lead to cellular toxicity and thereby to pathological phenotypes of RPE cells. Flatmount preparations of a variety of control mice showed a high specificity of Cre recombinase activation. Cre recombinase was only activated upon tamoxifen-treatment, confirming that the observed red fluorescence was due to recombinant expression and not a preparation artefact.

Analysis of the recombinant expression of tdTomato in the RPE revealed between 47.25% and 69.48% recombination in adult mice and 83.0% recombination in embryonic mice. Nonetheless, a percentage of unaffected cells may serve as internal controls and thus be beneficial in some circumstances. The difference of recombinant expression in adults vs. embryos could reflect differential dosing in the target tissue. Furthermore, recombination in the embryos may be more efficient, and effective since the RPE is actively developing as opposed to an already differentiated adult eye. Other studies have also observed differences when treating female vs. male mice with tamoxifen [26,27]. Therefore, it was unsurprising, that our treatment also led to different recombination levels in female (49.35%) vs. male (69.48%) mice. This is most likely caused by differences in the homeostasis of sex hormones, e.g. estrogens, especially considering that tamoxifen is an estrogen antagonist. We showed that Cre-mediated recombination in the *RPE-Tyrosinase-CreEr*^*T2*^ mice led to uniform distributions of expression patterns throughout the RPE monolayer as can be seen on the RPE flatmounts. This was statistically verified and analysis of the local distribution found no differences in expression between dorsal/ventral/nasal/temporal regions. However, we do see a difference in expression levels between the central vs. peripheral regions of adult RPE. This might reflect regional differences in the level of *Tyrosinase* gene expression. Since we still do not achieve a fully penetrant recombination, this may lie in the induction via tamoxifen and inefficient excision of the floxed exons. Expression of the *Tyr-GFP* construct shows virtually complete coverage, suggesting that the chosen promoter targets most cells in the RPE.

Compared to the existing non-inducible RPE-specific *Cre* mouse lines, *Dct-Cre* [8], *Tyrp1-Cre* [9], *MART1-Cre* [14], and *BEST1-Cre* [4], our *RPE-Tyrosinase-CreEr*^*T2*^ line has the benefit that it is possible to start recombination in both, embryonic and postnatal stages and thereby adds a temporal dimension for establishing gene control of RPE development and function. Since the onset of expression in those lines is at set time points (E12.5, E10.5, E12.5, and P10, respectively) having the flexibility of choosing the onset of gene recombination in embryos, neonates and adult mice will be of interest for studying the role of gene function in both, development and adulthood [4,14]. Furthermore, all other existing lines show ectopic expression. For gene knockout of widely expressed genes, knockout in the telencephalon (*Dct-Cre*), the skin (*MART1-Cre*) or testis (*BEST1-Cre*), could result in unintended side effects [4,8,14]. More relevant may be the ectopic expression in the neuroretina when using the *Tyrp1-Cre* line, which might be problematic when analyzing retinal phenotypes caused by loss-of-function mutations in the RPE [9]. In this regard, our newly generated *Cre* line had minimal ectopic expression. The expression in the retina was negligible (below the limit of quantification), confined to the INL, and was not cell type specific. In contrast to the reported inducible RPE-specific *Cre* lines, *Mct3-Cre* [15] and *VMD2-Cre* [5], our *RPE-Tyrosinase-CreEr*^*T2*^ mice show a higher level of recombination and an earlier possible onset of expression since the other genes are expressed later in RPE development. The *Mct3-Cre* line exhibits relatively low levels of recombination in adults (5–20%), whereas the recombination level in embryos was not reported [4,15]. The *VMD2-Cre* line shows the highest enzymatic activity at P4, whereas the level of recombination has not been reported so far [4,5].

Taken together, we generated a tamoxifen-inducible RPE-specific *Cre* transgenic mouse line, with high levels of uniformly distributed recombination in embryos and postnatal mice. This mouse line will serve as a valuable tool for those, who are interested in studying the functional role of gene expression in the RPE. This may ultimately be advantageous for development of new therapeutic targets to prevent RPE causative visual dysfunction.

## Supporting information

S1 Fig *Tyr-GFP* expression *in vivo*. Fluorescent image showing the high expression of GFP throughout an RPE flatmount of an adult *Tyr-GFP* mouse. Roman numerals and corresponding boxes indicate the regions which were taken for Fig 1B. Scale bar: 500 μm.
(TIF)

S2 Fig Absent Cre-mediated recombinant expression in control tissue. A: Representative image of an adult *Ai14;RPE-Tyrosinase-CreEr*^*T2*^ retina flatmount showing minimal tdTomato expression. Differential interference contrast (DIC) image was overlaid with the red fluorescent tdTomato expression image. B: Representative image of an adult *Ai14;RPE-Tyrosinase-CreEr*^*T2*^ RPE flatmount treated with β-Estradiol only. Outline of the RPE indicated by the solid line, dotted line represents inverted choroidal tissue. Scale bars: (A,B) 500 μm.
(TIF)

S3 Fig Cre-mediated ectopic expression in the retina is not cell type-specific. Representative immunofluorescence images of retina sections from treated adult *Ai14;RPE-Tyrosinase-CreEr*^*T2*^ mice, stained with antibodies against (A) Calbindin, (B) Calretinin, (C) PKC α, and (D) GS. Ectopic expression never co-localized with any of the inner retina specific markers. RPE: Retinal pigment epithelium, OS: Outer segments, IS: Inner segments, ONL: Outer nuclear layer, OPL: Outer plexiform layer, INL: Inner nuclear layer, IPL: Inner plexiform layer, GCL: Ganglion cell layer. PKC α: Protein kinase C α, GS: Glutamine synthetase. Scale bars: (A) 50 μm, (B-D) 75 μm.
(TIF)

S4 Fig Validation montage image as generated by the analysis program. After microscopy, the raw image was processed using color correction, contrast adjustment, and the background was manually removed, resulting in the processed image. Using this image, the program measured first the total area in pixels and afterwards the fluorescent cells in pixels. On the far right panel, the images for total area und measured cells were merged.
(TIF)

S5 Fig Representation of RPE locations used for regional comparisons. A: Example of RPE flatmount divided into dorsal (magenta) vs. ventral (cyan) and nasal (magenta) vs. temporal (cyan) areas. B: Example of RPE flatmount divided into central (cyan) vs. peripheral (magenta) areas.
(TIF)
