# Supplementary figures and images for: Generation of an inducible RPE-specific Cre transgenic-mouse line
Source: PLoS One. 2018 Nov 15;13(11):e0207222. doi: 10.1371/journal.pone.0207222 (PMC6237357; doi:10.1371/journal.pone.0207222)

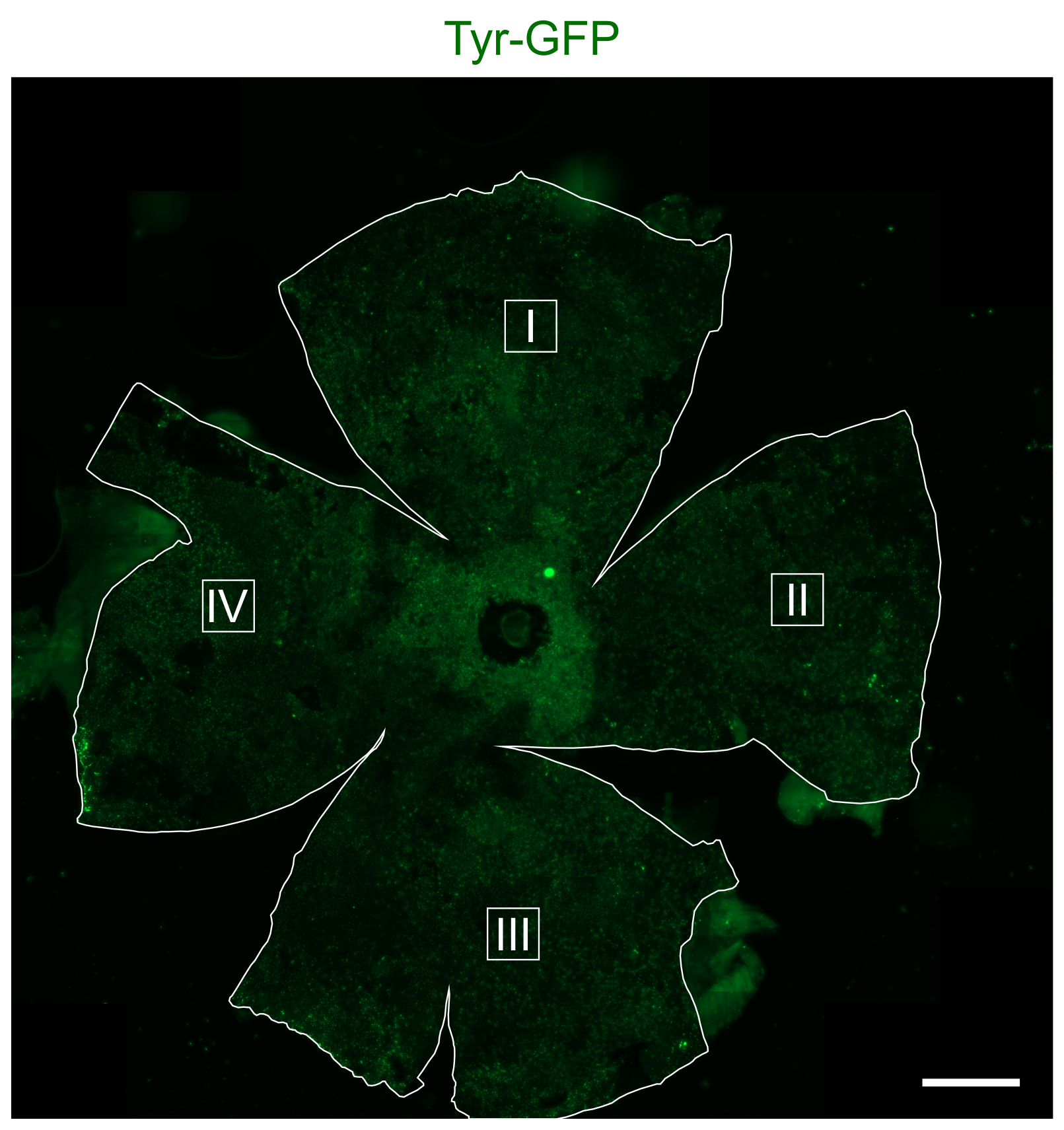

Supplement: S1 Fig — Fluorescent image showing the high expression of GFP throughout an RPE flatmount of an adult Tyr-GFP mouse. Roman numerals and corresponding boxes indicate the regions which were taken for Fig 1B. Scale bar: 500 μm. (TIF) [file pone.0207222.s001.tif]

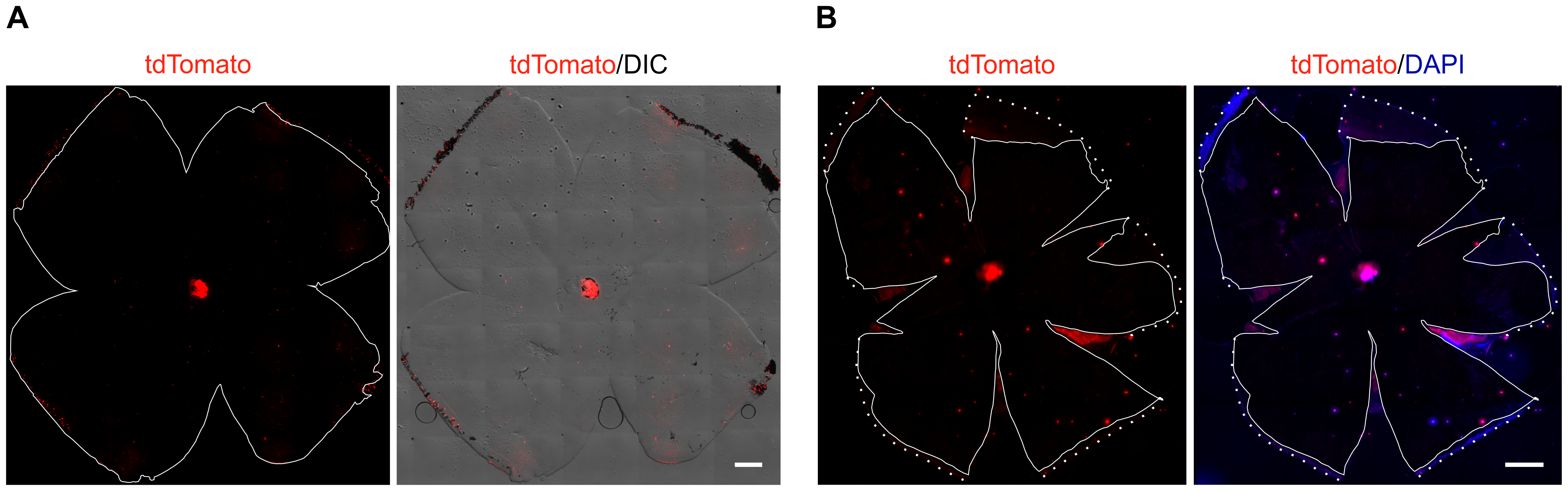

Supplement: S2 Fig — A: Representative image of an adult Ai14;RPE-Tyrosinase-CreErT2 retina flatmount showing minimal tdTomato expression. Differential interference contrast (DIC) image was overlaid with the red fluorescent tdTomato expression image. B: Representative image of an adult Ai14;RPE-Tyrosinase-CreErT2 RPE flatmount treated with β-Estradiol only. Outline of the RPE indicated by the solid line, dotted line represents inverted choroidal tissue. Scale bars: (A,B) 500 μm. (TIF) [file pone.0207222.s002.tif]

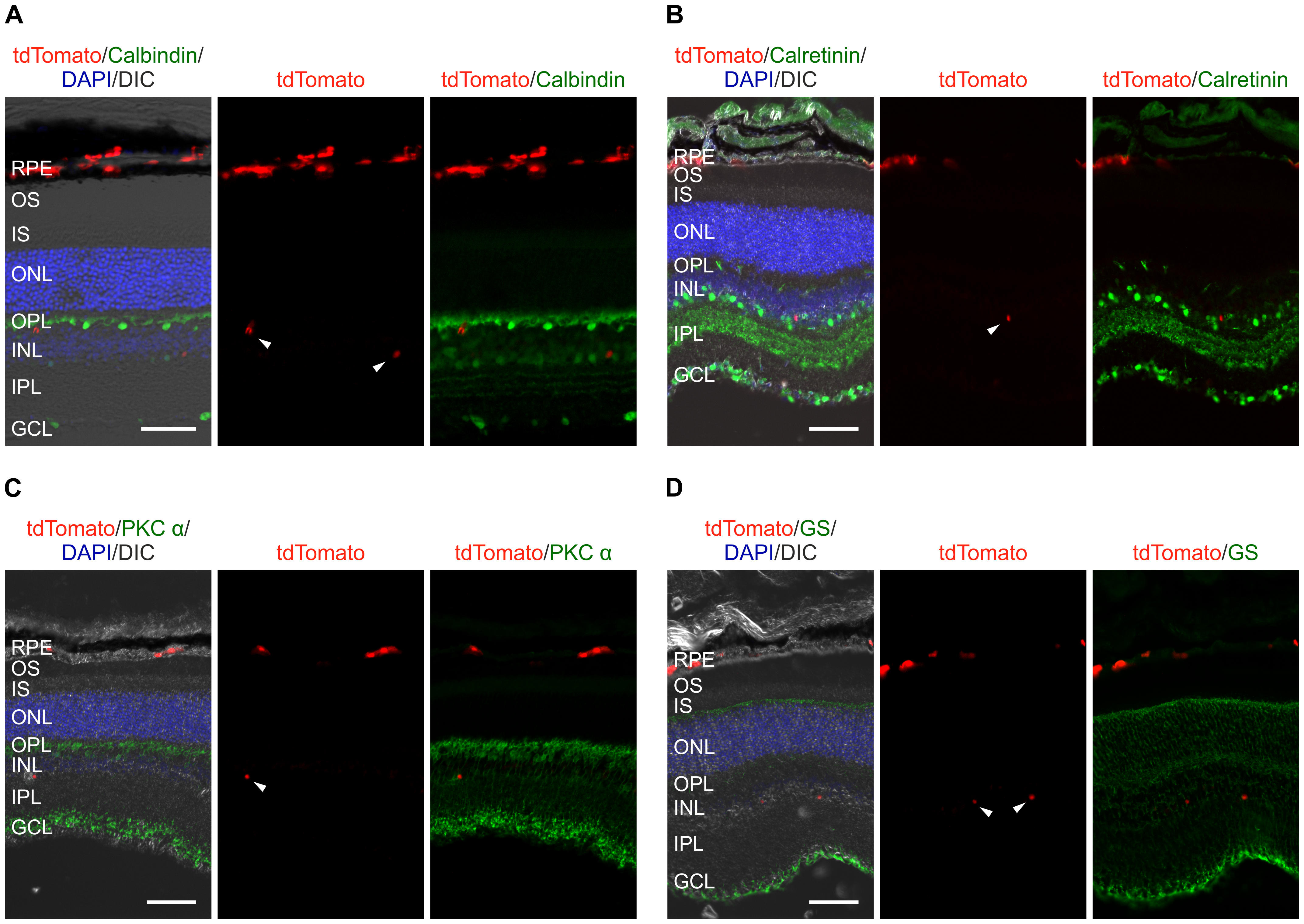

Supplement: S3 Fig — Representative immunofluorescence images of retina sections from treated adult Ai14;RPE-Tyrosinase-CreErT2 mice, stained with antibodies against (A) Calbindin, (B) Calretinin, (C) PKC α, and (D) GS. Ectopic expression never co-localized with any of the inner retina specific markers. RPE: Retinal pigment epithelium, OS: Outer segments, IS: Inner segments, ONL: Outer nuclear layer, OPL: Outer plexiform layer, INL: Inner nuclear layer, IPL: Inner plexiform layer, GCL: Ganglion cell layer. PKC α: Protein kinase C α, GS: Glutamine synthetase. Scale bars: (A) 50 μm, (B-D) 75 μm. (TIF) [file pone.0207222.s003.tif]

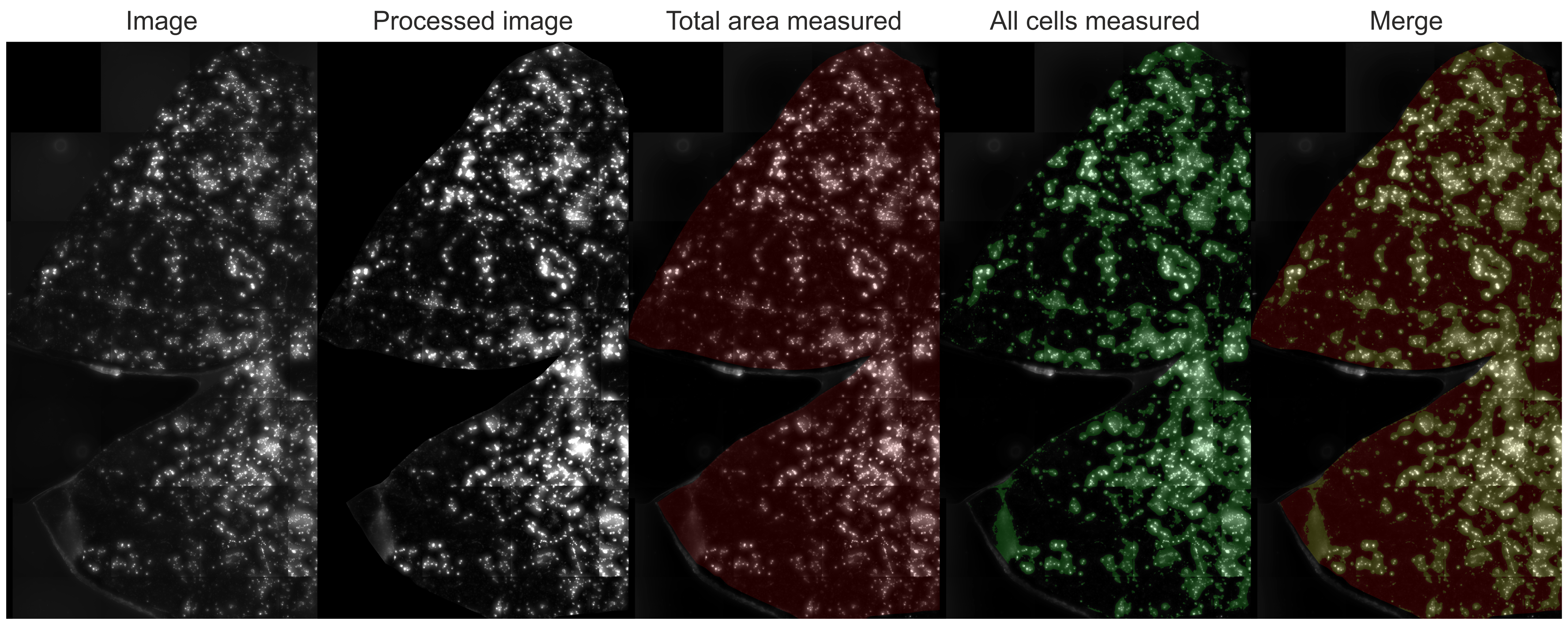

Supplement: S4 Fig — After microscopy, the raw image was processed using color correction, contrast adjustment, and the background was manually removed, resulting in the processed image. Using this image, the program measured first the total area in pixels and afterwards the fluorescent cells in pixels. On the far right panel, the images for total area und measured cells were merged. (TIF) [file pone.0207222.s004.tif]

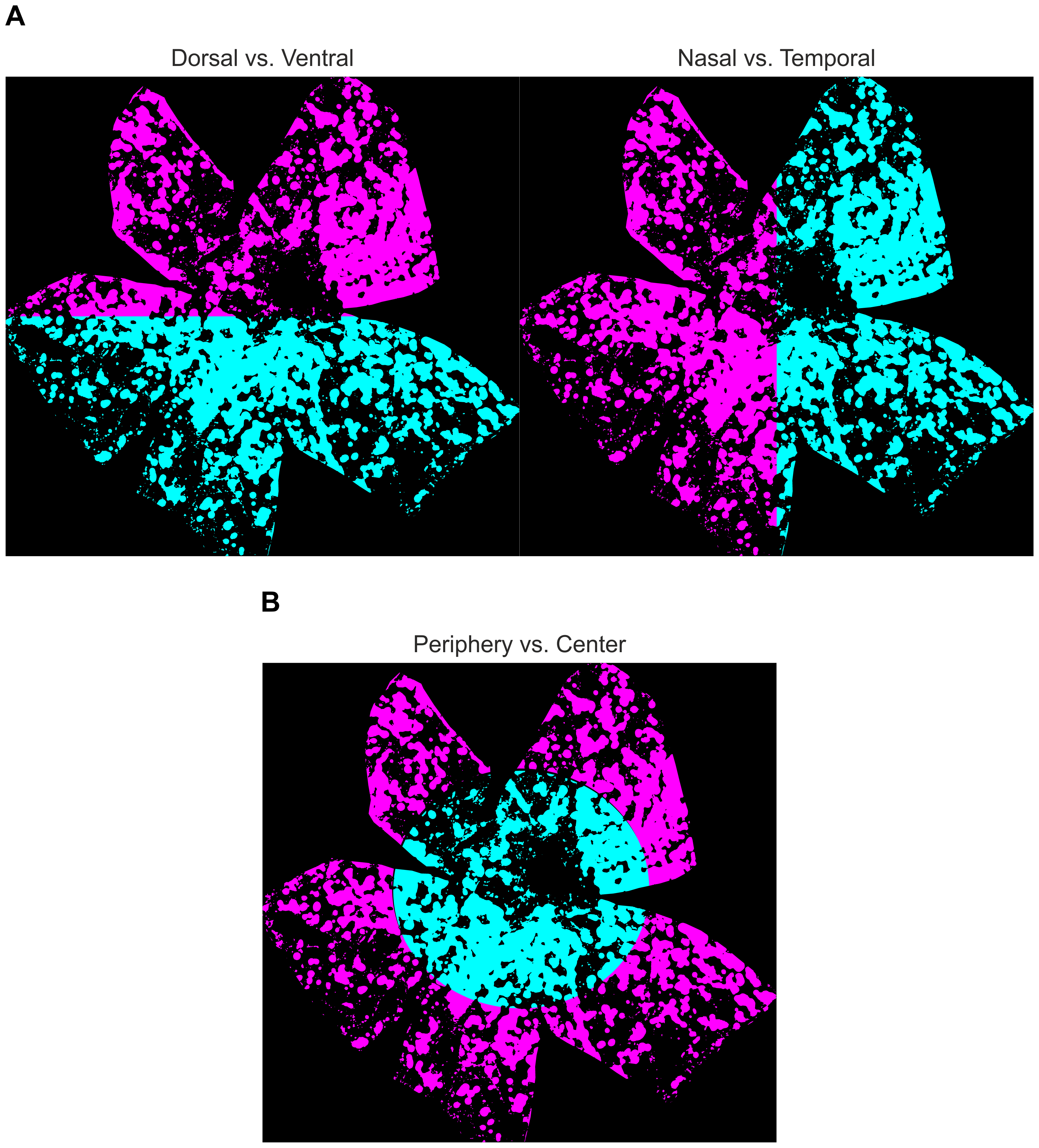

Supplement: S5 Fig — A: Example of RPE flatmount divided into dorsal (magenta) vs. ventral (cyan) and nasal (magenta) vs. temporal (cyan) areas. B: Example of RPE flatmount divided into central (cyan) vs. peripheral (magenta) areas. (TIF) [file pone.0207222.s005.tif]
